# Multi-Modal Social Robot Behavioural Alignment and Learning Outcomes in Mediated Child–Robot Interactions

**DOI:** 10.3390/biomimetics10010050

**Published:** 2025-01-14

**Authors:** Paul Baxter

**Affiliations:** School of Engineering and Physical Sciences, Lincoln Centre for Autonomous Systems, College of Health and Science, University of Lincoln, Lincoln LN6 7TS, UK; pbaxter@lincoln.ac.uk

**Keywords:** behavioural alignment, child–robot interaction, human–robot interaction, interactive activation and competition, learning effects, Sandtray, social robotics, touchscreen-mediated interactions

## Abstract

With the increasing application of robots in human-centred environments, there is increasing motivation for incorporating some degree of human-like social competences. Fields such as psychology and cognitive science not only provide guidance on the types of behaviour that could and should be exhibited by the robots, they may also indicate the manner in which these behaviours can be achieved. The domain of social child–robot interaction (sCRI) provides a number of challenges and opportunities in this regard; the application to an educational context allows child-learning outcomes to be characterised as a result of robot social behaviours. One such social behaviour that is readily (and unconsciously) used by humans is behavioural alignment, in which the behaviours expressed by one person adapts to that of their interaction partner, and vice versa. In this paper, the role that robot non-verbal behavioural alignment for their interaction partner can play in the facilitation of learning outcomes for the child is examined. This behavioural alignment is facilitated by a human memory-inspired learning algorithm that adapts in real-time over the course of an interaction. A large touchscreen is employed as a mediating device between a child and a robot. Collaborative sCRI is emphasised, with the touchscreen providing a common set of interaction affordances for both child and robot. The results show that an adaptive robot is capable of engaging in behavioural alignment, and indicate that this leads to greater learning gains for the children. This study demonstrates the specific contribution that behavioural alignment makes in improving learning outcomes for children when employed by social robot interaction partners in educational contexts.

## 1. Introduction

As robotics applications become more pervasive and desired in a wide variety of human-centred environments, it has become necessary to consider social interaction between humans and robotic devices [1]. Such consideration is not only necessary for those robots with humanoid-inspired morphologies (for which there may be an explicit emphasis on social interaction), but also those robotic systems that take on a more ‘industrial’ style (for example, automated transport vehicles operating in warehouses where people are also working). Endowing robots with some degree of human-like social competence can ease interactions around and with people. This can allow people to interact with robots in a manner more similar to interacting with other humans, thereby potentially reducing the need for specialist training, for example. Relevant social competencies in this context go beyond overt and conscious interactive behaviours (such as spoken conversation) to also include both non-verbal behaviour, extending to those behaviours that are employed subconsciously.

There remain numerous technical challenges in the deployment of robots to human-centred environments, including those related to perception and navigation in highly dynamic and unpredictable contexts. With respect to social interaction though, an important source of inspiration (and indeed technical guidance, as we see in this paper) is the study of humans themselves from a wide range of perspectives. In the most general sense, this is because humans provide the range of desired behaviours that we may wish to approximate and deploy on robots. The sources of this inspiration include psychology, cognitive science, etc., as well as the study of human behaviour and performance in specific contexts, such as collaborative manufacturing [2] and education [3]. These can provide a point of reference in the development of ‘appropriate’ (The notion of ‘appropriate’ here would be highly dependent on application context, etc. Here, the term is used to indicate an acceptable (if not optimal) behavioural response to a situation in which a human (or multiple humans) is co-present.) robot behaviours, given the range of circumstances to which they may be exposed. Such behaviours include those employed automatically (and perhaps subconsciously) by people in social interactions. Indeed, given the importance of such relatively subtle behavioural cues in social interaction, these aspects can be of particular interest in social robot development, e.g., [4,5].

One such relatively subtle but pervasive social adaptation evident in human behaviour is behavioural alignment, which is proposed as a fundamental characteristic of social interaction [6]. This has been well characterised in terms of linguistic alignment in human verbal interactions, e.g., [7], but there is also evidence that such alignment is evident across interactive modalities [8]. Moving further from human–human studies, there is also evidence that alignment (and particularly linguistic alignment) is also present when people interact with computer devices [9,10]. As such an apparently fundamental social competence, it is one that is of interest to this present work: can it be observed, and if so, what impact does it have?

When considering social behaviour generation for autonomous robots, it is necessary to consider how such behaviour could be generated, i.e., what behaviours to exhibit, in what way, and at what time. As noted above, and in this context, inspiration can be sought from human cognitive processing. A range of cognitive architectures have been applied to HRI, e.g., [11], to address these issues. In our work, we take a similar approach, in particular, basing our learning models on inspiration from human memory [12]. This is specifically focused on associative networks and learning, with a specific emphasis on learning during ongoing social interactions, rather than pre-training algorithms offline and prior to deployment. This approach allows for adaptation to individuals in real-time, and enables an exploration of such adaptation to any outcomes of the social interaction of interest. Indeed, this is one of the aims of the present paper: facilitating behavioural alignment in social robot interactions using a biologically–inspired memory-based system.

In this paper, we seek to apply our memory-inspired adaptive system to support the behavioural alignment of a social robot towards a human interaction partner. The application domain of specific interest here is education, specifically primary school education, and we are thus concerned with social Child–Robot Interaction (CRI). In this context, social robots have been demonstrated to lead to improved learning outcomes for the children concerned, partly due to their physical embodiment and presence [13], although there is a lack of discussion of underlying theory (educational and pedagogical) [14]. The precise nature of all aspects of behaviour that contribute, or at least influence, this beneficial effect remains however a more open question. This is not least due to the contributors of human tutor and human learning partner behaviour not being completely characterised either. The application of our question of the impact of behavioural alignment to this domain therefore has a number of interesting facets that can be explored. Firstly, the presence of behavioural alignment between children and robots can be characterised. Secondly, the implications of this alignment (should it be present, though the literature suggests is it likely, cf. Section 4) for learning outcomes can be assessed. And thirdly, the principle of the application of our biologically inspired approach to behavioural adaptation can be evaluated in a real-world context.

The structure of the paper is as follows. The first section of this paper (Section 2) is dedicated to reviewing the motivations and technical basis of *touchscreen mediators*. A touchscreen provides the medium on which the educational content that is to be learned by the children is displayed, and provides a focus and context for the interaction. This touchscreen is also crucial to understand the nature and mechanisms of behavioural alignment that is employed in this contribution. This is followed in Section 3 by a description of the memory-based learning system that provides the substrate of the behavioural adaptation of interest. With these two foundations established (touchscreen interaction mediators and a memory-based learning system), a study is presented that intends to assess the relationship between behavioural alignment and learning outcomes in Section 4, with the results of this study presented in Section 5, including a discussion of a set of metrics appropriate for the analysis. Overall, we seek to demonstrate that behavioural alignment is present, and that it appears to support increased learning outcomes.

## 2. Touchscreen-Mediated Human–Robot Interactions

Our prior work in child–robot interaction (CRI) has relied on the use of a touchscreen that acts as a mediator between the child and the robot [15]—which we have termed the ‘Sandtray’—which was inspired by mediated interactions in child therapeutic interventions, e.g., [16]. Such a use of touchscreens and similar tabletop interfaces as mediators have also found applications in autism therapy and research: for example, Ref. [17] had a projector-based tabletop interface for fostering collaboration in autistic children, and Ref. [18] which was also collaborative, and used an interaction space on a projected surface.

Touchscreens (and tablet computers) have seen extensive use in human–robot interaction studies in the years since, as they present a versatile addition to the social robotics technologies themselves. Some notable examples (this being by no means an exhaustive list) include [19] with a collaborative game-playing robot using a touchscreen as a shared resource; Ref. [20] which indicated that the presence of a tablet resulted in fewer positive facial expressions compared to when the tablet was not there; and Ref. [21] using a tablet as a writing medium for both robot and child in a (demonstrably successful) handwriting development application scenario.

The purpose of this section is two-fold. Firstly, it is to motivate and introduce the technologies underlying the use of a touchscreen for mediating social interactions between robots and children—an aspect that is generally neglected in the literature. This is necessary to contextualise the interactions that take place in the user study that is presented later in this contribution (Section 4), both in terms of technical implementation details, and the impact it has on social interaction dynamics and robot behaviour development. The second purpose is to describe the use of the touchscreen as a virtual modality for the robot, both in terms of perception and action, and how this is taken advantage of in the presented study.

### 2.1. Mitigating Technical Issues: Focusing on the Interaction

In the social interaction context we consider in the present work, there are three significant technical advantages to employing touchscreens in the manner described. First is the simplification provided in terms of sensory processing. When interacting with real objects in the real world, robot control systems require a high-level of processing of visual data in order to provide position and identity information (e.g., where is the object of interest and what is the object of interest). Since all the ‘virtual objects’ on the screen are placed there deliberately (by the control programme employed), their position and identity can be accessed directly by the robot control system, thus avoiding some of this sensory-processing complexity. Related to this is the ability of the touchscreen to provide additional information about the behaviour of the human, through their interaction with the touchscreen itself. This type of information is readily available (interpreted as mouse clicks for example), and provides a similar reduction in the need for complex vision-based processing in order to detect when and how the human interacts with the virtual objects. It is this facet in particular that we take advantage of in the study presented later in this contribution.

The second benefit to using touchscreens as mediating devices is the simplification in robot motor control required for the manipulation of the virtual objects on the screen. As with perception, the robot control system has the potential to directly access the location, behaviour, and identity of the virtual objects displayed on the screen. Instead of a computationally costly and mechanically difficult manipulation task, relatively straightforward robot orientation behaviours (e.g., pointing) can be combined with direct manipulation of the virtual objects to achieve the same effect.

A third benefit of using such touchscreens is the implicit constraints it places on the interacting human, with the resulting advantages for the robot and its internal/external sensory apparatus. In a setup where the human and robot face each other over the Sandtray, there is a limitation in the position that the human could be in order to comfortably interact. This implicit constraint eases potential problems for sensory systems, as some assumptions can be made regarding the position of the human (e.g., face tracking, knowing that the human, if present, is likely to be in front of the robot).

Naturally, these technical problems are not insurmountable, and there is an impressive array of research solutions under development for each of these. What we are attempting to highlight, however, is that by reducing computational complexity, the application of touchscreens allows the focus of system development efforts to be on the social behaviour of the robot itself, rather than the (in this context) supporting technologies.

In addition to this, we suggest that there is a distinct advantage to the use of such a mediating touchscreen in terms of the social interaction itself. We have previously shown that in child–child interactions mediated by the same type of touchscreen, the content of the interaction is constrained by the task provided on the screen (i.e., the children would talk about the task provided): there is an implicit constraining effect apparent [22]. In the context of human–robot interaction, this restriction on the contents of the interaction can provide a significant advantage in technical terms. For example, dialogue management in truly open interactions remains a significant challenge due to the difficulty in appropriate utterance planning when there are few, if any, limitations. Given the implicit structuring that the presence of the touchscreen-based task facilitates, the dialogue managements systems can be constrained to task-relevant information. Similarly, open speech recognition remains a challenging technical issue, particularly where children are concerned [23]. As with dialogue management, the implicit constraints facilitate a focusing of the technical system—enabling, for example, the more effective application of keyword-based recognition, which can frequently attain high performance (though in perhaps more constrained contexts).

### 2.2. Robot Interaction with the Touchscreen

**Figure 1:**
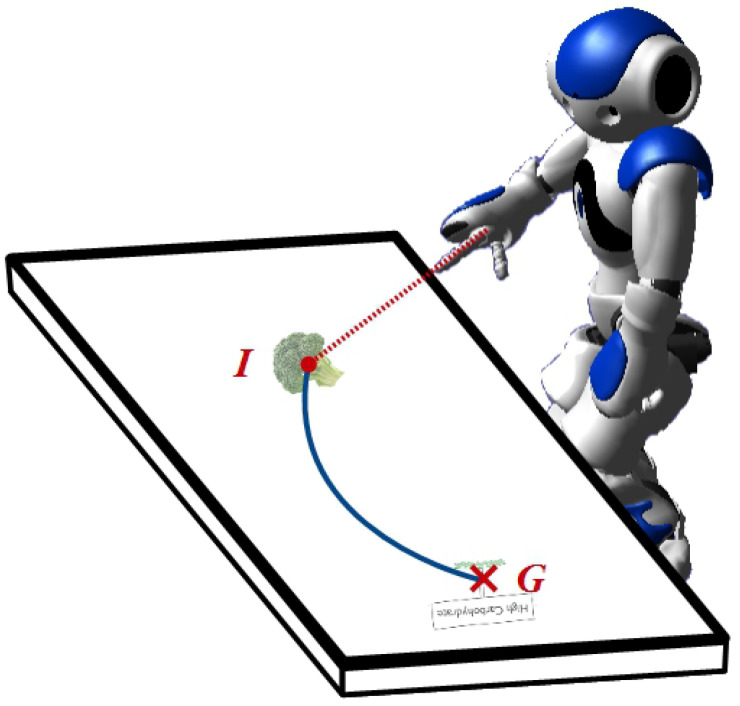
A humanoid robot (Aldebaran Nao is shown) interacting with a virtual object displayed on the touchscreen: in the example interaction shown, an image is to be moved from an initial position (I) to a goal position (G), along a defined path (blue line). The robot does not make direct contact with the screen, but instead coordinates its movement with that of the virtual object shown on screen.

As described above, one of the primary benefits of using touchscreens in the manner proposed is that it can act as a *virtual modality* for the robot. For example, because all of the objects displayed on the touchscreen are controlled by the server, the robot control system can be passed this information directly, without the requirment of complex vision processing (Figure 1). This process assumes that the geometric arrangement of the robot and touchscreen is known so that the spatial relationship between the robot and the objects displayed on the touchscreen can be directly calculated using a straightforward coordinate transformation. This spatial arrangement calibration can, of course, be automated based on visual processes (e.g. marker placement and recognition); however, for the purposes of the present context, we consider this to be a separate issue.

In a similar manner, robot movements can be coordinated directly with the virtual objects, without the robot needing to manipulate the screen directly (Figure 1). For the robot we are using, and indeed for the majority of humanoid robots available for social human–robot interactions, a direct manipulation of the touchscreen using the robot hands is not a feasible option; firstly because the materials used are not conducive to detection using capacitive touch sensors, and secondly due to the unreliability of the fine motor control required to maintain contact between the robotic device and the touchscreen surface. To circumvent these issues, a virtual pointing and dragging control system is used, whereby the position of the virtual objects displayed on the screen is synchronised with the movement of the robot arm (e.g., Figure 1). Note that with our pointing behaviours, we also use the head direction: i.e., the robot head is oriented in the same direction as the arm is pointing.

#### 2.2.1. The Sufficiency of Robot-Pointing Behaviours

The effectiveness of the robot–touchscreen interaction is dependent on the appropriate interpretation of the robot-pointing behaviour. This is naturally dependent on the morphology of the robot used. In our work, we exclusively employ the Nao small humanoid robot (Aldebaran/Softbank Robotics/United Robotics, e.g., Figure 1). Their appearance lends them to child–robot interaction; however, their manual dexterity is severely limited. In addition to this, the two-fingered and large hand setup does not afford a straightforward interpretation of pointing direction.

Despite this, a number of studies have shown that the Nao’s pointing direction is actually interpretable to a high degree of consistency. With a Nao and the human subjects standing next to one another, one study demonstrated more than 99% comprehension of robot-pointing direction [24]. With the robot across from a shared workspace, as in our configuration, a similar effect is observed, with gaze requiring pointing in order to be reliably interpreted, although the role of speech cues is also emphasised [25]. In addition, our own observations over an extended number of studies with children show that they, in the context of a touchscreen-mediated collaborative task, will readily accept that the robot is manipulating objects on the screen.

In the case of other robots, the manual dexterity limitations of the Nao may be more readily overcome. Nevertheless, the grasping and manipulation of real objects is typically not the focus of research in social HRI—except where this aspect is under specific investigation. As such, the role of a touchscreen as described here remains a valuable resource in facilitating the focus on social interaction aspects (Section 2.1).

#### 2.2.2. Bezier Curve-Based Movements

There are a number of possible approaches when it comes to the robot interacting with the touchscreen. From the perspective of interaction affordances, there are only a limited range of possible robot actions that would be reasonably expected, combinations of which can serve a range of functions: pointing and ‘dragging’, with either one or both hands/arms (of a humanoid-like robot morphology).

(1) $$\begin{array}{cc} \mathrm{Where}\;t & \in [0,1]\\ \mathrm{and}\;\vec{p}\left(t\right) & =(x_{t},y_{t})\\ \vec{p}\left(t\right) & =\vec{a}t^{3}+\vec{b}t^{2}+\vec{c}t+p_{I}\\ \mathrm{where}\;\vec{c} & =3*(p_{a}-p_{I})\\ \vec{b} & =3*\left(p_{b}-p_{a}\right)-\vec{c}\\ \vec{a} & =p_{G}-p_{I}-\vec{c}-\vec{b} \end{array}$$

The most straightforward means of implementing this would be straight-line movements from an initial position to a target location. However, to achieve a more naturalistic motion, we employ a path-planning algorithm based on cubic Bezier curves, a procedure commonly used in computer graphics applications, e.g., [26]. The procedure is as follows: given the initial ($p_{I}$) and goal ($p_{G}$) points of the desired on-screen movement (for example, to move a particular food object to one of the category locations), two control points ($p_{a}$ and $p_{b}$) are defined, and a Bezier curve is constructed (resolving for both x and y dimensions; see Equation (1)).

**Figure 2:**
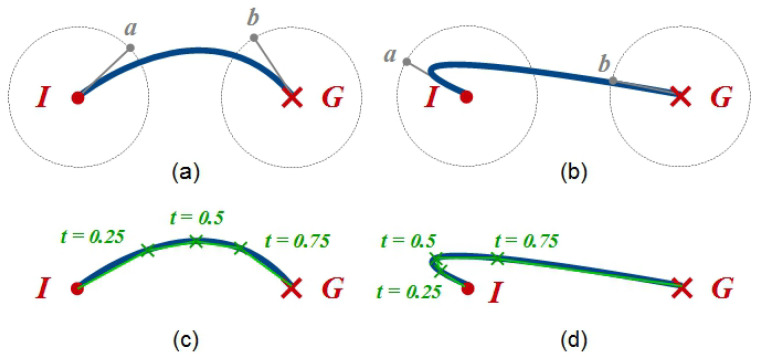
Constructing Bezier curve paths for virtual objects: the control points *a* and *b* are defined on the circumference of a unit radius circle around the initial (*I*) and goal (*G*) points of the movement, respectively. (**a**) Standard curve; (**b**) Bezier curve giving the impression of the robot ‘changing its mind’ on goal location mid-move by setting the first Bezier control point in the opposite direction to the goal location; (**c**) converting Bezier curve to robot control behaviour using three intermediate Bezier curve parameter values; (**d**) intermediate points are closer together on sharp curves facilitating robot control.

The control points ($p_{a}$ and $p_{b}$) are chosen randomly for each movement on a unit radius circle around the start and end points to allow for variability in each of the movements (Figure 2a). As the parameter *t* in the calculation of $\vec{p}$ increases from 0 to 1, the value of $\vec{p}\left(t\right)$ returns a coordinate along the Bezier curve. The nature of Bezier curves means that given a linear distribution of equal partitions of *t*, a non-linear distribution of points along the Bezier curve results, with relatively more points occurring where the rate of change in curvature is greatest (e.g., Figure 2c). For the robot-pointing control system, we use intermediate points at three values of *t*: 0.25, 0.5, and 0.75. A linear interpolation is used between the five point sequence (start, three intermediate, and end) for the robot movement. The on-screen virtual object movement uses the same Bezier curve.

The timing of the screen and robot moves are also synchronised. The use of Bezier curves, and the linear interpolation using three intermediate points (Figure 2c,d), provides a ready means of achieving this, since the path curvature is taken into account by the non-linear distribution of intermediate points (t∈{0.25,0.5,0.75}). Computing the length of the linear path between intermediate points (segment) as a proportion of the total path length gives the proportion of time (of the total movement time) available for the segment. The total time available is itself determined from the desired speed of movement, which in the present study is one of the variables adapted by the robot itself (cf. Section 4.4).

A further consequence of this application of Bezier curves is that the trajectory used by the robot to move the target virtual object to a categorisation location is not always easy to predict. For example, if the first control point ($p_{1}$) is chosen in the opposite direction to the goal location, then the robot will first appear to move the virtual object away from the target before moving towards it (Figure 2b). This may give the impression to the child participants that the robot is ‘changing’ its mind, despite the pre-planned nature of the movement. Thus, while the robot does not make physical contact with the screen at any point, the non-linear movements of both robot and virtual objects facilitate the illusion of the robot control. Indeed, in the numerous trials run with children (a number of which are reported in this article), the issue of how the robot controls the touchscreen only seldom arises, indicating the effectiveness of the approach.

### 2.3. The Touchscreen as a Virtual Modality

In summary, the involvement of a touchscreen in a human–robot interaction reduces the practical problems that arise with robot perception and control in the real world. In this context, the touchscreen effectively constitutes a ‘virtual modality’ for the robot, directly providing perceptual information and the capacity to physically interact without either increasing the computational load on the robot, or exceeding its physical constraints. By employing a reliable technical solution such as this, the focus of the robot behaviour and sensory apparatus can be oriented towards the human interactant rather than the task itself, lending it to social HRI applications.

Employing such a virtual modality facilitates the adaptation of the behaviour for the robot and perception of human touchscreen-oriented behaviours. All the motions are parametrised (e.g., using the Bezier curves, Section 2.2.2) for both the robot movements and those of the objects displayed on the touchscreen in a reliable manner. Furthermore, the characterisation of the child touchscreen-oriented behaviour is simplified, also leading to a reliable source of information. It is on this basis that we employ our adaptive robot behaviour; what remains is the mechanism through which such adaptive behaviour can be generated as informed by the behaviours of the human interaction partner. This is the subject of the following Section 3.

## 3. Behavioural Alignment Through Adaptive Associative Networks

A number of theoretical frameworks grounded in neuroscientific data have emphasised the distributed nature of human brain in terms of structure and function, e.g., [27,28]. Translating this perspective to synthetic systems—such as cognitive architectures—entails fundamentally reconsidering the role and nature of memory, and rather than viewing it as passive storage, instead treating it as a fundamentally distributed and associative active component of cognition that is the substrate over which activation dynamics equates to ‘cognitive processing’ [12]. The Distributed Associative Interactive Memory (DAIM) system has been developed to embody these core principles. The system is mechanistically an extension of classic Interactive Activation and Competition models [29], similar in part to other robotics-oriented implementations, such as ERA [30], in the application of Hebbian-like learning, but differing in not proposing any hubs of connectivity, leaving the structure to be informed by the ‘experience’ of the network.

While DAIM was initially applied to modelling the acquisition of concept prototypes to provide a developmental account [31], the intention behind its development was to help support naturalistic learning and behaviour on the part of social robots. As such, in this section, the core mechanisms of DAIM are described and how it is applied to the task of supporting behavioural alignment. First, the manner in which DAIM links to the virtual modalities of the touchscreen mediator is described (Section 3.1). Then, the activation dynamics (Section 3.2) and learning mechanisms (Section 3.3) are described, followed by an explanation for how robot behaviour can subsequently be derived.

### 3.1. Touchscreen Modalities

**Figure 3:**
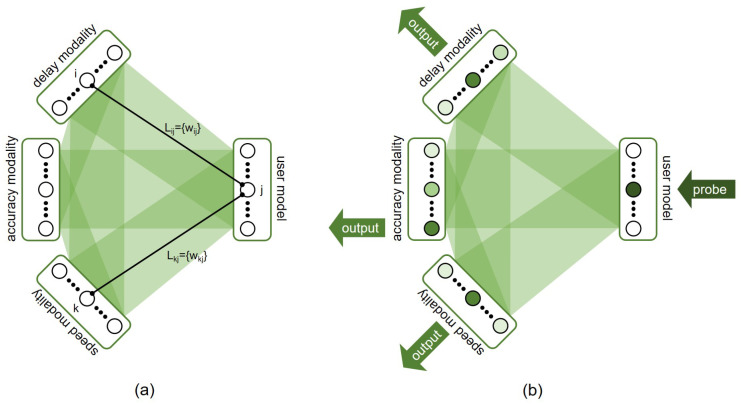
Connection structure of DAIM network used in the present study. (**a**) Three touchscreen-oriented modalities (delay, accuracy, and speed) are used, each constituting a modality. In addition to this, a user model modality serves to bind multi-modal information. Network structure is developed and adapted through the interaction (shaded regions indicate relationships between modalities in which associative links, e.g., $L_{ij}$, can form: note that all modalities can form associative links with all others). (**b**) Robot move parameters (in the adaptive condition) are obtained by probing the user model and reading out the units with the highest activation level in each of the modalities.

In previous work, an online-adaptive associative network to recognising touchscreen-directed human behaviour was applied. It was shown that the characteristics of individuals could be modelled, even over short interaction times [32]. This, however, was based on recorded data and offline training. In the present study, this same system—DAIM—is applied to real online interactions. Three touchscreen ‘virtual’ modalities are used to characterise touchscreen-directed behaviours, as outlined above (Section 2.3): delay between moves, accuracy of moves, and speed of moves. In addition to this, a *user model* modality is used that enables information from different modalities to be linked to an individual human interactant. The structure of the resultant network is shown in Figure 3. This structure enables relationships to be learned during an interaction, but also allows for the network to be probed to derive movements parameters for the robot.

At the start of an interaction, the network is empty: i.e., the modalities are present, but there are no associative links in the system. Through interaction, and specifically as the human performs touchscreen-oriented behaviour, associative links are formed between the individual modalities and the user model of the person present at that time (Figure 3a). Note that such associative links are also formed between the touchscreen modalities themselves—this supports the mechanism of cross-modal priming [33] (these links are not shown in Figure 3a for clarity). This process of formation and the subsequent updating of associative links allows the learning of the human touchscreen-oriented behaviour.

The DAIM system is essentially an Interactive Activation and Competition (IAC) network extended to incorporate online associative network structure generation and weight adaptation (learning). As such, there are two primary processes involved: flow of activation through the associative network (interactive activation and competition), and formation and update of associative weights (learning and adaptation). These processes occur in real-time as applied in the present study.

### 3.2. Activation Dynamics

A DAIM network is made up of a number of modalities (named ‘pools’ in the IAC convention), each of which are made up of a number of nodes. All nodes in the associative network have an *activation value*, which is a bounded scalar (between $a_{min}=-0.2$ and $a_{max}=1.0$), updated in discrete time, on every time-step:

(2) $$a_{i}=a_{i}+\Delta a_{i}$$

The activation of a node $a_{i}$ is influenced by three sources: prior activation of itself ($a_{i}^{prior}$ subject to a proportional decay $\delta_{g}=0.2$ towards a resting activation of $a_{rest}=-0.1$), activation from other nodes within the network it has associations with ($a_{i}^{int}$), and excitatory input from external sources ($a_{i}^{ext}$). The change in activation of every node ($\Delta a_{i}$) is determined by the following:

(3) $$\begin{array}{cc} If\left(a_{i}^{net}>0\right):\Delta a_{i} & =a_{i}^{net}\left(a_{max}-a_{i}\right)-\delta_{g}\left(a_{i}^{prior}-a_{rest}\right)\\ else:\Delta a_{i} & =a_{i}^{net}\left(a_{i}-a_{min}\right)-\delta_{g}\left(a_{i}^{prior}-a_{rest}\right) \end{array}$$

where $a_{i}^{net}$ is derived as follows:

(4) $$a_{i}^{net}=\left(\xi_{g}\times a_{i}^{ext}\right)+\left(\zeta_{g}\times a_{i}^{int}\right)$$

In Equation (4), two bias parameters are present to vary the influence the effect of internally derived activation ($\zeta_{g}=0.3$) and externally sourced activation ($\xi_{g}=0.6$). These are constant global parameters. In turn, these internal ($a_{i}^{int}$) and external ($a_{i}^{ext}$) sources of activation are derived as follows:

(5) $$a_{i}^{int}=\sum_{j=1}^{j=j_{max}}a_{j}\times w_{ij}$$

(6) $$a_{i}^{ext}\in \left[0,1\right]$$

where there are $j_{max}$ nodes with an associative link to node *i*, where each of these associative links have a weight $w_{ij}$, which is a bounded scalar value ([−1,1]). This external activation is driven by a process external to the system, such as a sensory value (for example, a discrete value for touchscreen move speed; see Section 4.4 for a full list of modalities and nodes used).

### 3.3. Associative Learning

The second mechanism of DAIM is the network structure generation and weight adaptation. At system initialisation, there are no associative links ($L_{ij}$, linking nodes *i* and *j*). Nodes may create a new associative link with any node, as long as they are located in a different modality to themselves: no within-modality associations are permitted. A new link of this kind (initialised with a weight $w_{init}=0.2$) is created *iff*:

(7) $$\mathrm{New}\;\;L_{ij}\;\mathrm{iff}\;\;\left(a_{i}>0.0\right)\cap \left(a_{j}>0.0\right)$$

On every time-step in the operation of DAIM, the weights of all existing associative links may be updated subject to a min/max saturation and learning rate ($\lambda_{l}$) and a mechanism to prevent a gradual weight increase given the negative resting activation level ($a_{rest}$) of nodes, as follows:

(8) $$\Delta w_{ij}=\left\{\begin{array}{cc} 0.0, & \mathrm{if}\;a_{i}\cap a_{j}=-0.1\\ \lambda_{l}a_{i}a_{j}\left(1-w_{ij}\right), & \mathrm{if}\;a_{i}a_{j}>0.0\\ \lambda_{l}a_{i}a_{j}\left(1+w_{ij}\right), & \mathrm{otherwise} \end{array}\right.$$

After which the weight is simply updated as follows:

(9) $$w_{ij}=w_{ij}+\Delta w_{ij}$$

To account for the unpredictable nature of real sensory data, additional mechanisms have been incorporated into DAIM. This ensures that only those weights connecting nodes between two modalities (say, *A* and *B*) that have an external activation input ($a_{A}^{ext}$ and $a_{B}^{ext}$) greater than some threshold ($A_{T}=0.1$) are updated. This implements a data-driven dependency to learning, rather than allowing learning to be driven purely by internal activation dynamics. The parameter manipulated is the learning rate ($\lambda_{l}$, Equation (8)), which may thus be different ($\lambda_{high}=0.01$, $\lambda_{low}=0.001$) between each pair of modalities:

(10) $$\lambda_{l}=\left\{\begin{array}{cc} \lambda_{high}, & \mathrm{if}\;a_{A}^{ext}\cap a_{B}^{ext}>A_{T}\\ \lambda_{low}, & \mathrm{otherwise} \end{array}\right.$$

This mechanism ensures that latent activation in the associative network does not cause an update of weights, if in the absence of externally driven activation. This latent activation remains an influence if this externally driven activation is present, thus maintaining the influence of activation dynamics on ongoing processing.

In order for the robot to make use of this learned information, the network can be *probed* (Figure 3b). This entails injecting activation into the relevant user model, and waiting for activation to spread through the associative network (since there is only activation input into one modality in this phase, minimal learning in the network takes place, as per Equation (10)). When activation settles in the individual modalities, the result is an activation profile across each modality. The parameters for the robot’s behaviour can then be derived directly from this profile (Figure 3b). In this way, the associative network both learns about the human interaction partners behaviour and is used to determine the robot’s behaviour: a common substrate for learning and action. This mechanism underlies the adaptive behaviours used in the study reported in Section 4.

## 4. Study: Impact of Touchscreen Supported Behavioural Alignment

The educational context is an ideal domain in which to study social robots and in which some of the potential benefits to people can be demonstrated. The learner/robot interaction context provides a highly dynamic scenario, in which learning outcomes (which are observed for the children in such studies) are but one of the considerations [34], with special education providing further considerations and opportunities [35]. As noted above, the physical presence of robots in this educational context appears to be associated with learning gains on the part of the interacting children [13].

An evaluation study was conducted with three objectives. First, to illustrate how touchscreen-mediation can provide a basis and context for social human-robot interactions. Second, to show how using the touchscreen as a virtual modality (see above) provides information that can be directly used to inform robot behaviour. Set in the context of applying robots as social companions to facilitate learning by children, we thirdly seek to show the benefits of using touchscreen-derived information for robot behaviour on such learning, and in particular, assess the impact of the robot behavioural adaptation.

### 4.1. Research Questions

It is well known from human–human interaction studies that people will naturally align linguistically to one another through interaction, e.g., [7]. With sensorimotor communication forming an essential part of our suite of communication abilities [36], this effect of alignment has also been shown in humans across multiple modalities simultaneously [8], indicating that it is a fundamental mechanism that we as humans employ. Linguistic alignment in particular is also observed when humans interact with computers [9,10]. Similarly, studies with human–robot interaction have demonstrated that some degree of alignment to a robot’s behaviour will occur over the course of an interaction [37].

Given that here we are concerned with the touchscreen-oriented behaviour of the child and robot, and that behavioural alignment seems so fundamental to social interaction, we seek to establish whether, and to what extent, alignment to a robot’s behaviour will occur in the context of a touchcsreen-mediated interaction. Furthermore, given that humans appear to have a natural tendency to align their behaviour anyway (as outlined above), we wish to establish whether adaptivity on the part of the robot (i.e., allowing mutual alignment) can influence this interaction. Finally, we are interested in seeing whether such behavioural alignment on the part of the robot has any impact on the amount that the child will learn when exposed to touchscreen-centred task material displayed.

This leads to the three experimental hypotheses of this study:

**H1:**  Children will align their touchscreen-directed behaviours to those of the robot.

**H2:**  A robot that adapts its behaviour towards that of an interacting human using the touchscreen-directed modalities will lead to greater mutual alignment.

**H3:**  A human interacting with a robot that aligns its behaviour to that of themselves (adaptive condition) will learn more from the collaborative game than if interacting with a non-adaptive robot (baseline condition).

### 4.2. Experimental Setup

**Figure 4:**
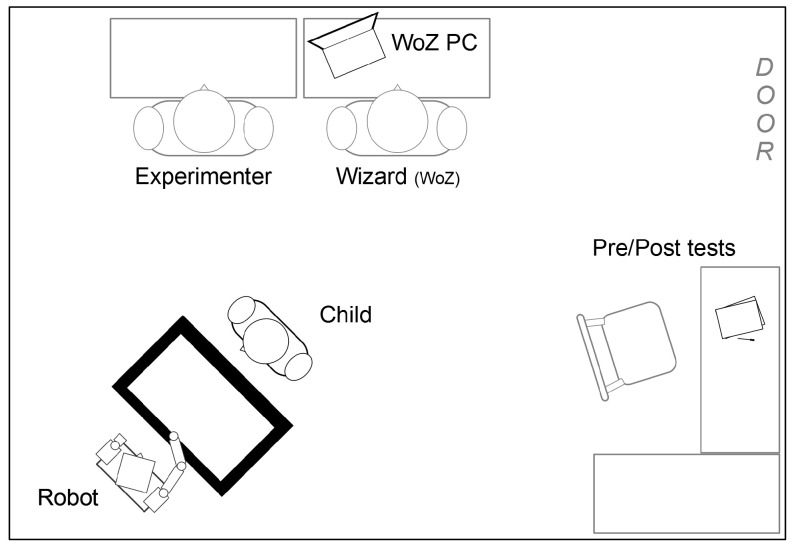
Typical setup of the experiment room: the experimenter and wizard remained out of direct line of sight during an interaction. The pre- and post-tests were completed in the same room as the interactions took place. Not to scale.

The study was conducted with children in three UK primary schools (all in year 3, therefore aged 7–8 years). The environment in which the study took place was therefore familiar to the respective children; not being a laboratory setting reduced the degree of control over experiment variables, but offers the opportunity to observe participant behaviour in a more ecologically valid environment. A total of 26 children took part in the study (N=26, Table 1), each of whom engaged in a one-on-one interaction with the robot in a room separate from the normal classroom, but one with which the children were familiar. The experiment room varied in size across the three schools (in terms of dimensions and contents), but the setup used was the same: a Nao robot, a ‘Sandtray’ touchscreen device, two video cameras, one experimenter, and one ‘wizard’ operator (Figure 4).

The experimenter was responsible for bringing the children to the experiment room from their classroom, briefing the children on what would happen, administering the paper-based pre-test, introducing the robot system (as a peer), administering the post-test, and then debriefing the children whilst returning them to their classroom. During this process, the children were out of their classroom for between 15 and 20 min. The variation in this time out of the classroom was primarily due the speed with which the children filled in the pre- and post-tests. This process was not rushed, and the children were free to take their time. The role of the wizard was to initialise the robot system, start the robot behaviour at the start of the interaction, and initiate the end of interaction behaviour after 5 min of interaction time. Each of these behaviours, once triggered, ran autonomously. All of these functions were controlled through a GUI on a remote PC. During the interactions, both the experimenter and wizard remained in the experiment room, but were consistently positioned to be out of the direct line of sight for the child (Figure 4).

To investigate the hypotheses of the study, two experimental conditions were employed: the Adaptive condition (‘A’), in which the robot would adapt its behaviour towards that displayed by the child towards the touchscreen, and a Baseline (control) condition (‘B’), in which the robot would not display such an adaptation, and not vary its behaviour through the interaction. Children were randomly assigned to the two conditions, while maintaining gender balance (Table 1).

### 4.3. Experimental Protocol

Each individual interaction consisted of the following. The child was brought into the room and asked to complete a 14-item paper-based quiz to assess their knowledge of carbohydrates, emphasising that guessing was acceptable if the answer was not known. One of two versions of the paper-based quiz were administered, randomly assigned to individuals but balanced across conditions, to control for possible differences in difficulty or prior knowledge between the quizzes. It is shown below in the results that there is an equivalent difficulty (see Section 5.2).

**Figure 5:**
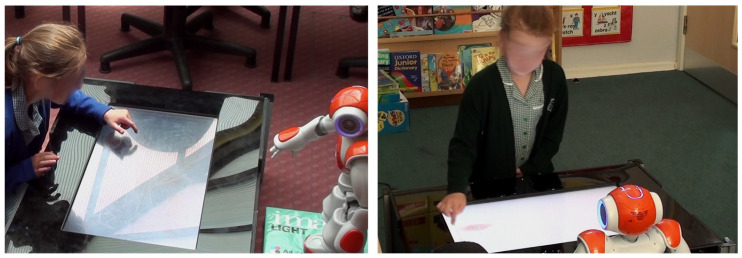
Two sample interactions showing how the children and robot collaboratively interacted around the touchscreen.

**Figure 6:**
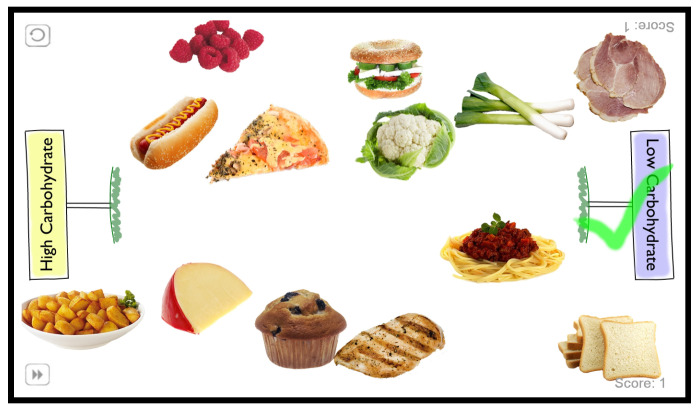
Example set of food images shown on the touchscreen during the sorting task with the two categories used (low and high carbohydrate content). Visual and auditory feedback is given upon classification events: the green tick shown denotes a recent correct classification.

The child was then brought over to the robot, at which point the experimenter explained to both the child and the robot what the task was; the robot behaviour was autonomous with a human wizard (out of direct line of sight of the child) triggering the different phases of the behaviour (initialise, start, and end). The task was a sorting game played collaboratively between the robot and subject on the Sandtray touchscreen (Figure 5): displayed were a number of images of food, with the task being to decide whether each type of food had lots or only a little amount of carbohydrate contained (Figure 6). Both the subject and robot could make moves on the screen: the screen displayed visual and auditory feedback on a classification event by either interactant (for both correct and incorrect classifications). The robot did not explicitly employ a turn-taking strategy, although in most cases, such a behaviour emerged [38]. Once all the images had been classified, a new set of food images could be displayed, with the category positions reversed on alternate image sets. Images of food were divided into sets only to minimise the overlap of images on the screen at any one time. The robot acted as a peer throughout the interaction; this meant that the robot made mistakes (to varying degrees, see below), and encouraged rather than instructed with its feedback utterances.

At the end of a five minute period of gameplay, the robot would announce that it was tired (a phase initiated by the wizard), at which point the experimenter would intervene, ask the subject to complete a post-interaction quiz (the version not completed as a pre-test), and return the child to the class with the request that they did not discuss the robot or task with their classmates until the end of the day. Subsequent reports from the respective class teachers indicated that the children all conformed with this request.

### 4.4. Robot Behaviours

As noted above, two types of robot behaviour conditions were employed, depending on the experimental condition. In the baseline condition, the robot would perform classification moves at a set rate, with a set probability of being correct (50%) and with a set speed of movement. In the adaptive condition, the only difference was the adaptive modification of these movement parameters depending on the behaviour of the interacting subject, as learned by the associative-learning mechanism (DAIM) detailed in Section 3, and characterised in [32]. The differences in the robot behaviour between the conditions is summarised below (Table 2): each of the touchscreen modalities (classification %, move speed, and move delay) constitutes a ‘modality’ used by the associative-learning mechanism (Section 3). In this way, the robot’s speed of movement, accuracy of classification, and delay between moves was varied depending on how the subject behaved in these aspects. Robot utterances (introductions, in-game feedback, and closing), timings (other than delay between moves), etc., in both conditions were identical. As such, the differences between the conditions are in accordance with the primary hypotheses of the study (namely **H1** and **H2**), even though they could be seen as relatively subtle.

## 5. Results

The results are divided into three parts. In the first, the touchscreen-directed behaviour of the child and robot is analysed, particularly with respect to the alignment of the two for the evaluation of hypotheses **H1** and **H2**. In the second, the learning outcomes are assessed in relation to the experimental conditions, relevant to hypothesis **H3**. Finally, the association between the characterisation of touchscreen-directed behaviour and the learning outcomes is examined in the context of exploratory analysis.

In the results below, a test of normality was applied to all obtained sets of data. The Shapiro–Wilk test was employed as it provides the most powerful test of normality given all types of distribution and sample size, although the sample sizes used in the present study mean that this power is somewhat reduced [39]. Unless otherwise stated, the data were found to be consistent with a normal distribution according to the Shapiro–Wilk test.

Given the normality of data, and given that hypothesis testing based on p-values is an unreliable measure, confidence intervals (CIs) provide a more accurate means of characterising the variability of the effect [40]. Following this, the results below provide 95% CIs in an attempt to provide a more complete perspective on the magnitudes and relative importance of the effects [41], rather than solely reporting on the outcome of null-hypothesis significance tests.

We furthermore provide the outcome of a bootstrapping process on the learning gain results. Bootstrapping is employed to provide estimations of population hypothesis testing from our collected samples [42], which does not assume any particular statistical distribution. We use $10^{6}$ replications with the *basic* bootstrap 95% CI reported, given our large replication pool [43].

### 5.1. Behavioural Alignment

As described above (Section 4.4), three aspects of the robot screen-directed behaviour were the subject of experimental manipulation: movement speed, accuracy of move (in terms of success of classification), and time delay between moves. These three aspects are based on the equivalent behaviour of the interacting child. These are denoted as *modalities*. Taking the example of a single modality, alignment between the interacting partners can be observed if the observed values converge over time.

A single metric is required in order to characterise the degree to which convergent (or divergent) alignment takes place. This metric is calculated separately for each modality, which are then combined to form a single indicator of alignment for an individual: the Alignment Factor (AF). For each modality, we may first compare whether the difference in robot behaviour and the child behaviour decreases between the first third of the interaction and the final third of the interaction. This is appropriate in particular due to the relatively short interactions examined in the present study (five minutes), and is similar to a procedure previously used that characterised gaze over time in social HRI [44].

The first step requires finding the arithmetic mean of each modality $\bar{M_{n}^{s}}$ for both the robot (s=r) and human (s=h), in both the first (n=1) and final (n=3) thirds of the interaction (of total length *T*, Equation (11)). Each action in each modality for both the robot and human is represented by an ordered pair $p_{n}^{s}=\left(x^{s},t^{s}\right)$ (of which there are $m_{n}$ instances from the total set of actions $P^{s}$) comprising a value ($x^{s}$) and a time of occurrence ($t^{s}$).

(11) $$\begin{array}{cc} \bar{M_{n}^{s}} & =\frac{\sum x^{s}\in \left\{p_{n}^{s}\subseteq P^{s}\right\}}{m_{n}},\\ \mathrm{where}:\;n & =\left\{\begin{array}{cc} 1, & \mathrm{if}\;t^{s}\in \left\{0,...,\frac{T}{3}\right\}\\ 3, & \mathrm{if}\;t^{s}\in \left\{\frac{2T}{3},...,T\right\} \end{array}\right.,\;\mathrm{and}\;m_{n}=\left|p_{n}^{s}\subseteq P^{s}\right| \end{array}$$

From these values for both the human and robot in the interaction, the absolute difference between them can be derived for the first ($\delta_{1}^{m}$) and final ($\delta_{3}^{m}$) thirds of the interaction (Equation (12)). This difference remains modality specific, but does give an indication of whether alignment is likely to have taken place on an individual modality basis.

(12) $$\delta_{1}^{m}=|\left(\bar{M_{1}^{h}}-\bar{M_{1}^{r}}\right)|,\;\delta_{3}^{m}=\left|\left(\bar{M_{3}^{h}}-\bar{M_{3}^{r}}\right)\right|$$

**Figure 7:**
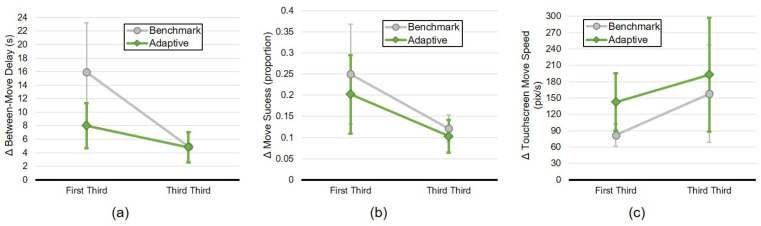
Mean difference between first and third thirds of the interaction, for each modality: (**a**) delay between touchscreen moves, (**b**) move success (classification), (**c**) touchscreen-oriented move speed. A convergence is seen both for delay and success rate, though there is a moderate divergence for move speed. Error bars show 95% CI.

Comparing these values, it is possible to visualise the presence (or not) of an alignment effect for each of the modalities individually (Figure 7). For both move delay (Figure 7a) and move success (Figure 7b), alignment (i.e., convergence in values between the robot and human) is evident. An effect to be noted is that in both of these cases, the value for the adaptive condition is lower than that for the benchmark condition: this is likely due to the presence of mutual alignment, even during the first moments of the interaction. For the speed modality, divergence from first to final thirds of the interaction is apparent (Figure 7c), although this effect is more pronounced for the benchmark condition. Further inspection of the data reveals few significant results, but rather trends that support these observations (Table 3).

One further feature to be noted from these plots is that in both conditions, the degree of alignment is more similar between the conditions in the final third of the interaction than in the first third; a phenomenon that applies to all three modalities.

For each modality *m*, for interaction pair *i*, the Alignment Factor can then be calculated (Equation (13)). This is a dimensionless metric (i.e., independent of the individual modality scale), and takes a value in the range [−1,+1], where +1 is maximal convergence (i.e., alignment) and −1 is maximal divergence. A value of $AF_{m}=0$ (when $\delta_{1}^{m}=\delta_{3}^{m}$) indicates no change.

(13) $$AF_{m}^{i}=\frac{\left(\delta_{1}^{m}-\delta_{3}^{m}\right)}{\left(\delta_{1}^{m}+\delta_{3}^{m}\right)}$$

The overall alignment factor $AF^{i}$ for individual *i* is then simply the arithmetic mean of all individual modality alignment factors ($AF_{m}^{i}$, where $N_{m}$ is the total number of modalities considered, Equation (14)). This is similarly dimensionless, and based on the derivation of the factors for each modality (Equation (13)), it can be seen how this can incorporate any number of modalities.

(14) $$AF^{i}=\frac{\sum_{m=1}^{m=N_{m}}AF_{m}^{i}}{N_{m}}$$

**Figure 8:**
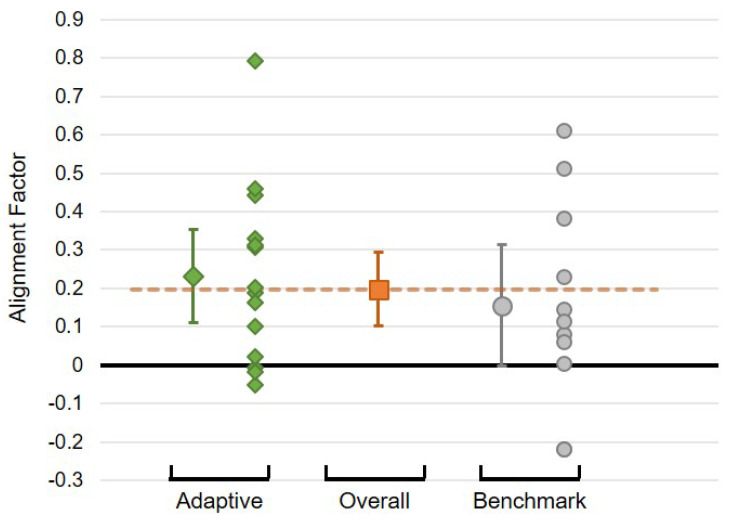
Summary of the Alignment Factors found for each individual child, split by condition: Adaptive and Benchmark. There is an overall alignment effect apparent (orange point/line), which is marginally higher in the Adaptive condition. Error bars are 95% CI.

Considering the values of the alignment factor obtained for each individual, it may be seen that there is a relatively high degree of variability within the conditions, and that there is relatively little difference (in the mean) between the conditions (Figure 8). Nevertheless, overall characteristics by condition can be derived: while there is no significant difference between the conditions (t(23)=0.763, p=0.453), there is an indication that Alignment Factors in the adaptive condition as a group are above zero (indicating alignment: 95% CI [0.110,0.353] does not contain zero), whereas this is not the case for the benchmark condition (95% CI [−0.002,0.313]). This result provides moderate support of hypothesis **H2** (a greater mutual adaptation in the adaptive condition than in the benchmark condition).

These results demonstrate moderate support for hypothesis **H1**, that children will align their behaviour to that of the robot. Considering only the benchmark condition, the robot does not adapt its behaviour based on that of the child. Any alignment apparent must therefore be due to the adaptation of the child’s behaviour with respect to that of the robot. This is clearly visible for both the delay and accuracy modalities, but not for the speed modality. The overall alignment factor for the benchmark condition is nevertheless positive (even though the 95% CI just includes zero), suggesting that some degree of alignment of the child’s behaviour towards that of the robot does occur.

### 5.2. Learning Outcomes

Two versions of the multiple-choice knowledge quiz were used to control for possible differences in difficulty (see Section 4.3). Both tests were used as pre-test and post-test, with this being assigned randomly to subjects, maintaining balance between conditions. Assuming that the quizzes were of equal difficulty, it would be expected that when used as a pre-test, there should be no difference between the scores (i.e., prior to learning). This was indeed found to be the case, with a 95% CI of the difference in the means of the pre-test scores for the two quizzes being [−1.856,3.856]. By including zero, this indicates that they can be considered as of equivalent difficulty. The learning results follow from this basis.

One further assessment of the validity of the results is to check whether the pre-test scores were equivalent between conditions: i.e., to check that children in both conditions started from the same point in terms of the knowledge to be learned. The 95% CI’s of the two test scores significantly overlap, though with the Benchmark performance exceeding that of the Adaptive condition (Adaptive: [4.518,7.482], Benchmark: [6.645,10.855]). We may thus say that the children in both conditions were matched in terms of ability. However, since the 95% CI of the difference in means does not include zero ([0.097,5.403]), as an additional control, we further employ the normalised learning gain metric to characterise learning outcomes, as this controls for pre-test score (see below).

The absolute increase in score from pre-test to post-test provides a preliminary indication of learning. Both the benchmark condition (mean: 0.833, 95% CI: [−1.079,2.746]) and the adaptive condition (mean: 2.5, 95% CI: [0.912,4.088]) demonstrate increases, although only in the adaptive condition is the 95% CI greater than zero. However, the 95% CI of the difference in the means of the two conditions suggests no significant difference between the conditions ([−0.928,4.262]).

However, a problem with characterising learning as the absolute increase in score from pre-test to post-test is that there is an upper bound to the possible score that could be achieved: for the present case, there is a maximum score of 14 achievable in both pre- and post-tests. Because of this, high pre-test scores will tend to result in lower absolute gains, leading to a negative correlation between the two [45]. One means of avoiding this issue is the application of the *normalised learning gain* metric introduced by [46], *g*:

(15) $$g=\frac{score_{post}-score_{pre}}{score_{max}-score_{pre}}$$

In contrast to absolute increase, normalised learning gain (Equation (15)) is uncorrelated with the pre-test score [46]. Because it normalises the learning outcome of the individuals based on their pre-test score, we employ *g* to characterise learning so that differing levels of prior knowledge are taken into account. This enables comparisons to be made across the subject group between the two conditions.

**Figure 9:**
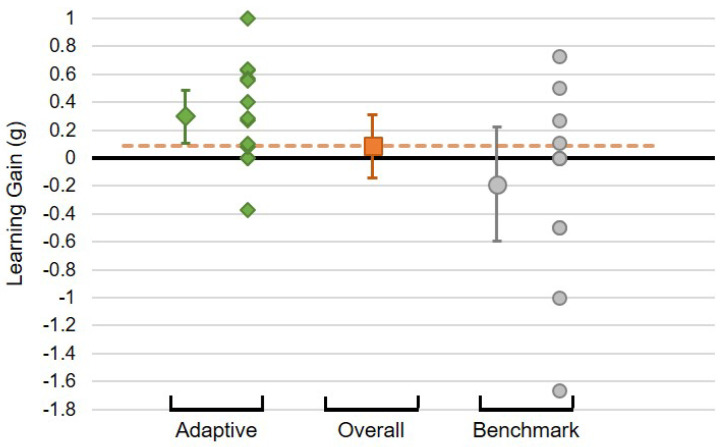
Mean learning gains of the Adaptive and Benchmark conditions. Orange point and dashed line: overall mean across both conditions. Error bars show 95% CI.

The mean normalised learning gains obtained by children in the two conditions may be seen in Figure 9: the non-adaptive benchmark condition contains zero ([−0.511,0.288]), whereas that of the adaptive condition is entirely above zero ([0.109,0.485]). Thus, while the difference between the conditions demonstrates only a trend to significance (95% CI of the difference in means: [−0.035,0.823]), individually, the benchmark condition does not suggest a positive learning effect, but the adaptive condition does. This provides support for the acceptance of hypothesis **H3**.

Additional support for these results is provided by bootstrapping, which is a random sampling method that can be used to estimate a sample distribution. In this case, it is employed to establish a non-parametric distribution for the bootstrapped difference in means, against which the observed difference in means can be compared. For learning gain, the result of this process (Table 4) indicates that there is a greater learning effect expected in the Adaptive condition than in the Benchmark condition.

### 5.3. Alignment and Learning

**Figure 10:**
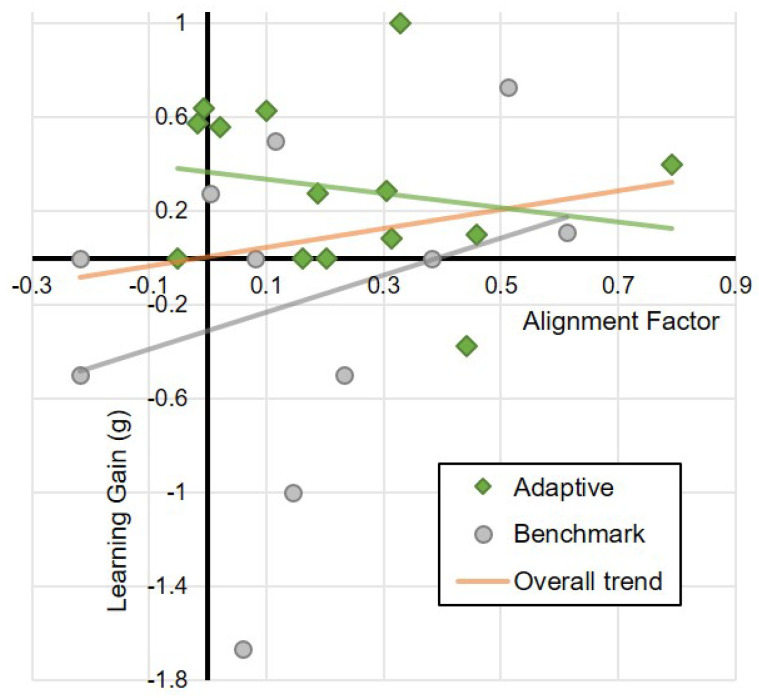
The relationship between Alignment Factor and mean normalised Learning Gain for the two conditions. Positive Alignment Factors indicate that the alignment of behaviours took place (over the three touchscreen-oriented behaviours examined); positive normalised learning gains indicate that learning took place. The orange line shows a linear trend line when both conditions are taken together, suggesting an overall positive correlation between the Alignment Factor and Learning Gain.

One final aspect of the results to consider is the relationship between the observed alignment factor and the learning gain achieved by the children (Figure 10). Since the learning outcome results seem to indicate that there is a significant learning effect for the Adaptive robot condition, it may be reasonable to expect that there would be a positive association between AF and learning gain. However, this is not observed as clearly as may be expected. While marginal effects may be interpreted from the graph, the slopes of the regression lines for both the Adaptive condition (95% CI: [−1.26,0.65]) and the Benchmark condition (95% CI: [−1.063,2.651]) are not significantly different from zero, indicating no meaningful association between learning gain and AF.

Following the discussion above, and given that there is some degree of alignment visible in both experimental conditions, considering both conditions together may provide an alternative perspective on the relationship between alignment and learning outcomes. A number of relationships are relevant to note (Table 5). As would be expected, there are strong positive correlations between the overall AF and the individual modality AFs (although the correlation with AF–delay is not statistically significant). What can be noted however is that the correlation between AF and learning gain (g), while positive, is weak (and statistically insignificant, regression slope 95% CI: [−0.59,1.39]), which reflects the discussion above (Figure 10). This positive (though not significant) effect may provide an initial indication that there is some impact of the degree of mutual alignment on learning gains, but there is insufficient data to support this directly.

Finally, we note that there are no significant correlations between boys and girls in pre-test scores (r=0.303,p=0.132) or in *g* (r=−0.230,p=0.258) across the conditions (n=26). While our experimental conditions were gender-balanced (Table 1), this indicates a lack of gender effects in this study: while there are known gender effects in educational contexts and in HRI, e.g., [47,48], these results justify our decision not to incorporate this aspect into the present experimental hypotheses, leaving such examinations to future work.

## 6. General Discussion

The study presented above was structured to assess three experimental hypotheses. In order to assess these, the notion of touchscreen mediators was discussed along with the technical details used in their implementation, and an associative-learning system was presented. Together, these allow our investigation of the presence and impact of robot behavioural alignment on child-learning outcomes.

The first experimental hypothesis (**H1**) proposes that children will align their touchscreen-directed behaviour to those of the robot. The benchmark experimental condition allows a direct examination of this, since the robot’s behaviour does not change over time; the child’s behaviour has no impact on the behaviour of the robot. While only a basic ratio metric, the alignment factor (AF) that was introduced characterises the degree of behavioural alignment for an individual interaction pair, in this case between the child and the robot. For the benchmark condition, it was shown that there is a mean AF of +0.155, indicating convergence between the touchscreen-directed behaviour of the child and the robot. This is not statistically significant (the 95% CI of the mean is [−0.002,0.313]), but shows a trend to a positive AF. As such, this indicates moderate support for H1. Having a positive AF for the benchmark condition is not necessarily surprising, given the range of existing observations in the literature regarding human behavioural alignment with a variety of computer and robot devices in a range of modalities (Section 4). In being in accordance with the existing literature, this result is nevertheless useful in demonstrating the robustness of the methodology we employ here.

The second experimental hypothesis (**H2**) extends H1 by suggesting that greater mutual alignment will be observed if the robot adapts its behaviour (in this case, aligns its behaviour towards that of the child), than if it does not. Considering the same set of results as for H1, but this time also considering the AF results for the adaptive condition, it may be seen that there is also marginal support for H2. In this case, while there is not a significant difference in AFs between the two experimental conditions, the mean alignment factor for the adaptive condition is significantly greater than zero (95% CI: [0.110,0.353]). As mentioned previously, given the observation from the literature and in relation to H1 that alignment will occur anyway on the part of the human involved, then the presence of increased mutual alignment when the robot also aligns its behaviour is not unexpected. What is established with this result though is the extent to which mutual alignment can be expected to occur in such an interaction scenario: i.e., the magnitude of the alignment effect. Looking more closely at the data in each modality (Section 5.1), it appears that one main source for the increased AF observed in the adaptive condition is that mutual behavioural alignment occurs faster in the adaptive condition than the benchmark condition; this seems to be correlated with improved performance in team interactions [49], which suggests that there could be further benefits to behavioural alignment beyond those explored in the present work.

The third experimental hypothesis (**H3**) focuses specifically on the impact on learning outcomes that alignment may be related to, with the proposal that greater learning outcomes will result from interaction with an adaptive robot than with the benchmark robot. Having established (H2) that there is greater mutual alignment present in the adaptive condition, the learning outcome results (as quantified using the learning gain metric to control for varying starting knowledge between participants) show that there is similarly a higher learning outcome in this adaptive condition, with this being statistically significantly greater than zero (95% CI: [0.109,0.485]). While there is no significant difference between the two experimental conditions, the learning gain observed in the benchmark condition is not significantly different from zero (95% CI: [−0.511,0.288]). We conjecture that the lack of significant difference between the conditions observed with respect to the learning gain are primarily a result of relatively small sample size, since the bootstrapping results support the conclusion of significantly increased learning outcomes in the adaptive condition. Taken together, the results support an acceptance of H3. In a separate work, we explored the effect of personalisation on learning outcomes, where non-verbal behavioural alignment was a key component of the personalisation strategy [50]. In that work, personalisation was found to have a significant and positive impact on the learning of new subjects by children. However, the notion of personalisation was a combination of multiple factors, including behavioural alignment and use of personal information. It is thus difficult to identify the relative contribution of each aspect to the learning outcomes. The present study provides some further insight to those results by providing an indication of what the contribution of alignment is to the overall personalisation effect and its consequences on learning. There remain further influencing factors to explore with respect to robot behaviour, e.g., the congruency of behaviours [51], but the present paper provides some further insight into the complexities of social human–robot interaction (in educational scenarios).

One result worth some further consideration regarding the correlations between AF and learning outcomes, as characterised by learning gain, is *g*. While it could be expected that given the presence of an apparent boost to learning outcomes in the presence of increased behavioural alignment (i.e., the adaptive condition) that there would be a positive association between AF and *g*, this is, however, not observed, with the correlation coefficients (as visualised in Figure 10) not significantly different from zero in either condition (and indeed with both combined). This result could be interpreted in a number of ways. Firstly, and possibly most likely in this case, it is possible that the sample size is simply too small for any effect size that may be present (unlike in the learning gain analysis, where the sample size with respect to effect size seems to be sufficient). A second possible interpretation is that the magnitude of the alignment (as characterised by AF) between the robot and child is not relevant, and it is the fact that mutual alignment is present at all that leads to the increase in learning gains observed previously. This is an intriguing possibility that the present study cannot directly address with the data collected, and so it must be left to future research. A third possibility is that there an intermediary effect at play that links behavioural alignment and learning outcomes that the current experimental methodology is not sufficiently sensitive to. For example, it is possible that the perception of the robot—in terms of social competencies and properties for example—differs significantly between the two experimental conditions. For instance, it is known that people will perceive a social robot as more or less intelligent based on its level of animacy [52], which is a behavioural property. Similarly, the attribution of anthropomorphic features vary based on the predictability of the behaviour of the robot [53]. To the extent that more human-like behaviour can be viewed as inherently more predictable (in the sense that they conform with humans’ expectation of how a social agent should behave), the adaptive condition may have resulted in a different social perception of the robot, which may have had consequences on the learning outcomes. However, without having collected the required data to resolve this (since the hypotheses did not cover this aspect), the present study cannot provide an answer to this question. Each of these possibilities, particularly the second and third points, require further empirical exploration to resolve.

In summary, the presented data show that a social robot is capable of engaging in non-verbal behavioural alignment, and indicates that increased mutual behavioural alignment leads to greater learning gains for the children. While mutual alignment is present even with a non-adaptive robot (the benchmark condition) due to the natural propensity of people to align to their interaction partners, the mutual alignment happens to a greater extent and faster when the robot also aligns its behaviour. In the present study, the alignment behaviour is supported by two systems: a mediating touchscreen that provides a set of virtual interaction modalities to the robot, and a human memory-inspired system (DAIM) that acts as an associative substrate for both learning and behaviour generation for the robot. This combination is well suited to the educational application context considered within this study, although there are some limitations to both in terms of extensibility if further applications are to be made (for example, in terms of simultaneous multi-party interactions, and the requirements of longer-term interactions). While further empirical investigation would be necessary to provide further insight into the precise mechanism(s) relating learning and alignment, this paper demonstrates the specific contribution that behavioural alignment makes in improving learning outcomes for children when employed by social robot interaction partners in educational contexts.

## Figures and Tables

**Table 1:** Child participants of the study by condition.

	Benchmark	Adaptive	Total
Boys	6	7	13
Girls	6	7	13
Total	12	14	26

**Table 2:** Summary of robot behaviour parameter differences between the two conditions.

	Benchmark	Adaptive
Classification %	50	[0, 100]
Move speed (pix/s)	400	[260, 540]
Delay between moves (s)	3.5	[2, 5]
Introduction	same	same
In-game feedback	same	same
Closing	same	same

**Table 3:** Comparing the first and final thirds of the interaction for each modality for both the adaptive and benchmark conditions. Mean for each modality shown (see Equation (11)), with sd (in brackets).

	Adaptive condition (n = 14)		
	1st	3rd	difference
Delay	7.999 (6.321)	4.819 (4.298)	t(26) = 1.557, *p* = 0.132
Accuracy	0.202 (0.178)	0.103 (0.075)	t(26) = 1.918. *p* = 0.066
Speed	142.562 (99.973)	192.793 (199.050)	t(26) = 0.844, *p* = 0.407
	Benchmark condition (n = 11)		
	1st	3rd	difference
Delay	15.932 (12.393)	4.913 (3.696)	t(20) = 2.826, *p* = 0.01
Accuracy	0.250 (0.200)	0.122 (0.053)	t(20) = 2.052, *p* = 0.054
Speed	81.988 (34.708)	157.917 (151.662)	t(20) = 1.619, *p* = 0.121

**Table 4:** Bootstrapping results for Alignment Factor and Learning Gain results. $10^{6}$ replications used; numbers in bold show observed values that lie outside of the bootstrapped 95% CI, which indicates a significant result.

Metric	Observed Difference of Means ($\bar{A}-\bar{B}$)	95% CI Bootstrapped Difference in Means
Alignment Factor (AF)	0.076	[−0.191,0.189]
Learning Gain (g)	**0.484**	[−0.440,0.450]

**Table 5:** Correlation coefficients for learning and alignment factor data, across both adaptive and benchmark conditions. Highlighted cells (bold) indicate significant correlations (at the p=0.05 level). *Increase*: absolute score increase (post-pre); *g*: learning gain; *AF*: alignment factor.

	Increase	g	AF	AF-Delay	AF-Accuracy	AF-Speed
increase	1					
g	**0.895**	1				
AF	0.092	0.172	1			
AF-delay	0.033	−0.116	0.234	1		
AF-accuracy	0.023	0.128	**0.486**	**−0.440**	1	
AF-speed	0.076	0.176	**0.646**	0.093	−0.155	1

## Data Availability

The raw data supporting the conclusions of this article will be made available by the authors on request. This applies to all anonymised data, but does not include images and/or videos: permission for sharing personalised data was not requested as part of the approved ethics application.
